# Isolation of Novel ACE-Inhibitory and Antioxidant Peptides from Quinoa Bran Albumin Assisted with an In Silico Approach: Characterization, In Vivo Antihypertension, and Molecular Docking

**DOI:** 10.3390/molecules24244562

**Published:** 2019-12-12

**Authors:** Yajun Zheng, Xian Wang, Yongliang Zhuang, Yan Li, Hailong Tian, Panqi Shi, Guifeng Li

**Affiliations:** 1 Food Science Institute, Shanxi Normal University, Linfen 041004, China; xssxsd2019@yeah.net (X.W.); tianhailong2019@yeah.net (H.T.); junqihan2015@yeah.net (G.L.); 2 Yunnan Institute of Food Safety, Kunming University of Science and Technology, Kunming, Yunnan 650500, China

**Keywords:** quinoa bran albumin, angiotensin-I converting enzyme, antioxidant peptides, inhibition kinetics, molecular docking, spontaneously hypertensive rats

## Abstract

Albumin is the major fraction of quinoa protein that is characterized as having high nutritional value. However, until now, scant information is available on the bioactivity of quinoa albumin or its hydrolysates. To promote its usage, we extracted albumin in this study from quinoa bran assisted with cellulase and hemicellulose, and hydrolyzed it by alcalase and trypsin to produce bioactive peptides. The hydrolysates (QBAH) were purified by gel filtration and reversed-phase high-performance liquid chromatography (RP-HPLC), followed by identification using liquid chromatography–mass spectrometry (LC-MS/MS). Furthermore, based on in silico analysis, one angiotensin-I converting enzyme (ACE)-inhibitory and antioxidant peptide, RGQVIYVL (946.6 Da), and two antioxidant peptides, ASPKPSSA (743.8 Da), and QFLLAGR (803.5 Da), from QBAH were synthesized. RGQVIYVL showed a high ACE-inhibitory activity (IC_50_ = 38.16 μM) with competitive mode of inhibition, and showed significant antihypertensive effect in spontaneously hypertensive rats at a concentration of 100–150 mg/kg body weight (bw). Molecular docking simulation showed that it could interact with the active ACE site via hydrogen bonds with high binding power. Moreover, RGQVIYVL, ASPKPSSA, and QFLLAGR all demonstrated high ·OH scavenging activity (IC_50_ = 61.69–117.46 μM), ABTS^+^ scavenging activity (58.29–74.28%) and Fe^2+^ chelating ability (32.54–82.48% at 0.5 mg/mL). They could also retain activity after gastrointestinal enzyme digestion. These results indicate that quinoa albumin is a potential source of bioactive peptides possessing antioxidant and ACE-inhibitory activities.

## 1. Introduction

In the recent past, food-derived bioactive peptides have attracted much attention for their potential to serve as natural alternatives or complements to synthetic drugs. These bioactive peptides are usually specific and small protein fragments (2–20 amino acids long) which are inactive within the parent protein but may reveal some functions such as antioxidant, antihypertension, anticancer, and antimicrobial activity after releasing from their protein sources [1]. Of these, peptides with angiotensin-I converting enzyme (ACE) inhibition and antioxidant activity have gained increasing interest over the last decade. It has been demonstrated that ACE makes a positive contribution to hypertension, which is the most common risk factor of heart diseases and threats to the life of one-fourth of adults in the world [2]. In renin–angiotensin system (RAS), ACE catalyzes the conversion of inactive angiotensin-I into angiotensin-II, a potent vasoconstrictor, and inactivates bradykinin, a potent vasodilator, resulting in an increase in blood pressure [3]. Thus, inhibition of ACE has become the main target in the treatment of hypertension. Moreover, oxidative stress can disturb the balance between oxidants and antioxidants in cells, leading to excessive free radicals, the balance being the important mediator of tissue injury in several neurodegenerative diseases and other pathological conditions [4]. Increasing epidemiological evidence has demonstrated that oxidative stress and associated oxidative damage contributed to the development and progression of hypertension and other chronic diseases such as arthritis, diabetes, and neurodegenerative diseases [5]. Furthermore, oxidation is also a major cause of food quality changes affecting the nutritional value, texture, and appearances and leading to undesirable off-flavors and potentially toxic reaction products. Hence, it is important to inhibit oxidation reaction and the formation of excessive free radicals occurring in the body. Both hypertension and oxidative stress have a critical link with diet; control and improvement of diet would be the easiest option to handle both risk factors [6]. To achieve this, the development of food-derived bioactive peptides with ACE-inhibitory and antioxidant activity would be a low-cost alternative to conventional synthetic therapeutics that always have possible harmful side effects. Until now, many studies have reported the purification, identification, and characterization of antioxidant and ACE inhibition peptides from animal proteins as well as plant and marine proteins. However, the number of studies focused on their in vivo antihypertension, structural bioinformatics, bioavailability, and action mechanism is limited [3,7,8,9,10,11]. 

Quinoa (*Chenopodium quinoa* Willd.) originating in South America is a gluten-free grain and has received increasing interest in other regions of the world for its high nutritional quality and high protein content (14–23%) [12]. It is widely grown in northwestern of China, especially in Shanxi Province, Gansu Province, and the Inner Mongolia Autonomous Region. Quinoa protein is characterized as having a balanced essential amino acid profile and being rich in lysine and methionine, which are the primary deficient amino acids in other cereals like rice, wheat, and maize [13]. The major proteins in quinoa seeds are albumins and globulins, accounting for 35% and 37%, respectively [11]. Recently, much attention has been given to the nutritional value and functional properties of quinoa protein [14,15], and bioactive peptides such as DPP-IV (dipeptidyl-peptidase IV) inhibitory peptides and antioxidant peptides have been identified from quinoa protein isolate and globulin [16,17,18,19]. However, scant information is available about the antioxidant and ACE-inhibitory activities of quinoa albumin or its hydrolysates. Quinoa bran is the byproduct of quinoa processing and a good source of protein and dietary fiber, but its usage in the food or other industries is limited [12]. Our previous study found that quinoa bran albumin hydrolysates demonstrated a considerable ACE-inhibitory activity (61.28% at 1.0 mg/mL) and hydroxyl radical scavenging ability (51.77% at 0.2 mg/mL), indicating that bioactive peptides with antioxidant and/or antihypertensive activity may be identified from it. Therefore, the present study focused on the isolation, characterization, inhibition kinetics, and in vivo antihypertension of ACE-inhibitory and antioxidant peptides from quinoa bran albumin. Moreover, the structure–activity relationship of the peptides was assessed through molecular docking simulation.

## 2. Materials and Methods

### 2.1. Materials

Quinoa bran was obtained from JingLe Yanmen Food Ltd. Co., XinZhou, Shanxi Province, China. N-Hippuryl-His-Leu hydrate (HHL) and ACE (from rabbit lung) were purchased from Sigma-Aldrich Co. (Louis, MO, USA). Ferrozine, cellulase (5.0 × 10^4^ U), hemicellulase (2.0 × 10^4^ U), alcalase (2.0 × 10^5^ U/g), pancreatin (1.0 × 10^5^ U/g), 2,2-azino-bis(3-ethylbenzothiazoline-6-sulphonic acid) diammonium salt (ABTS), and trypsin (1.0 × 10^5^ U/g) were purchased from Supei Biotech. Co., Ltd., Shanghai, China. Other chemicals and reagents were of analytical grade.

### 2.2. Extraction and Isolation of Quinoa Bran Albumin

Quinoa bran albumin extraction was carried out using the method described by Nwachukwu and Aluko [20] with some modifications. Briefly, the quinoa bran was defatted three times with *n*-hexane (1:10, g/mL), and then dried and passed through a sieve of 0.2 mm mesh. Afterward, 30 g of defatted quinoa powder were suspended in 300 mL of distilled water and adjusted to pH 4.5. Then, cellulase (50 U/g) and hemicellulase (50 U/g) were added and the mixture was incubated in a shaking water bath (SHA-B, Tianrui Instrument Co., Ltd., ChangZhou, China) at 45 °C for 1 h, and then adjusted to pH 9.0. After incubation at 40 °C for another 2 h, the mixture was heated at 100 °C for 5 min and filtrated. The residue was suspended in distilled water, adjusted to pH 9.0, and stirred at 40 °C for 2 h and then filtrated again. The filtration was pooled and centrifuged at 10,000× *g* for 30 min, and the resultant supernatant was collected and dialyzed against ultrapure water with a 3.5 kDa MWO dialysis membrane for 48 h at 4 °C. Then the dialysate was freeze-dried and recovered as albumin, and stored at −20 °C.

### 2.3. Preparation of Quinoa Bran Albumin Hydrolysates (QBAH)

Quinoa bran albumin was digested according to the method indicated by Chirinos et al. [9] with some modifications. Briefly, the quinoa bran albumin (dissolved in dH_2_O, 2 g/100 mL) was hydrolyzed by two enzymes in the following sequence—alcalase (45 °C, pH 9.0, 100 U/g protein, 1 h) and trypsin (37 °C, pH 7.0, 60 U/g protein, 30 min). The enzymes were heat inactivated in a water bath at 100 °C for 10 min. The supernatant was collected after centrifugation (10,000× *g*, 20 min) and lyophilized to obtain QBAH. The degree of hydrolysis (DH) was determined using Adler-Nissen’s method [21].

### 2.4. Angiotensin-I Converting Enzyme (ACE)-Inhibitory Activity and Inhibition Kinetics

The ACE-inhibitory activity was evaluated using HHL as a substrate by monitoring the released hippuric acid using a spectrophotometric method [22]. Briefly, the reaction system comprised 50 µL of ACE (25 mU), 150 µL of 8.3 mM HHL, and 50 µL of sample solutions. After incubation in a shaking water bath (37 °C) for 1 h, 1 M HCl (250 µL) was added to stop the reaction. The hippuric acid formed was subsequently extracted by 1.4 mL ethyl acetate. After centrifugation, 1 mL of the supernatant was collected and evaporated at 80 °C for 60 min in a vacuum. The residue was then dissolved in 2 mL of distilled water and the absorbance at 228 nm was read. The inhibitory activity was calculated as follows:

(1) $$\mathrm{ACE}\;\mathrm{inhibition}\;\left(\%\right)\;=\;\left(1-{\;A}_{S}/A_{C}\right)\;\times 100,$$

where A_C_ is the absorbance of the ACE solution without an inhibitor and A_S_ is the absorbance of the mixture-contained samples. The IC_50_ value, the inhibitor concentration required to inhibit half the ACE activity, was acquired by nonlinear regression from a plot of percentage ACE inhibition versus different inhibitor concentrations.

Lineweaver–Burk plots of 1/V versus 1/HHL in the presence of the inhibitor were used to analyze the ACE inhibition kinetics of the peptides identified in QBAH. The ACE enzyme activity was measured with various HHL concentrations (0.76, 1.52, 3.04, 3.80 and 7.60 mM) and peptide concentrations (0, 0.1, 0.2 and 0.5 mg/mL).

### 2.5. Scavenging Activity of Hydroxyl Radical

Hydroxyl radical (·OH) scavenging activity was assayed using the 2-deoxyribose oxidation method [23]. IC_50_ was defined as the concentration of peptide that was required to scavenge 50% of radical activity. The activity was determined using the following equation:

(2) $$\mathrm{OH}\;\mathrm{scavenging}\;\mathrm{activity}\;\left(\%\right){=\;[\;1-\;(A}_{S}{\;-\;A\;}_{B}{\;)/\;A}_{C}\;]\;\times 100,$$

where A_B_ is the absorbance of the blank (distilled water instead of samples), A_C_ is the absorbance of the control (without the addition of 2-deoxyribose oxidation), and A_S_ is the absorbance of the mixture-contained samples.

### 2.6. Purification by Gel Chromatography and Reversed-Phase High-Performance Liquid Chromatography (RP-HPLC)

QBAH (2 mg/mL) was filtered through 0.22 µm filter and subjected to gel chromatography on a Sephadex G-25 (Yuanye Biotechnology Co., Ltd., Shanghai, China) column (Φ 1.2 × 100 cm) eluted with distilled water at 2.8 mL/min and monitored at 220 nm. Each fraction was pooled, freeze-dried, and subjected to ACE-inhibitory activity and ·OH scavenging activity testing. The fraction with the highest activity was further purified by RP-HPLC on a Zorbax semi-preparative C_18_ column (SB-300, Φ 9.4 × 250 mm, Agilent Technologies, Palo Alto, CA, USA). Elution was eluted at a flow rate of 2.2 mL/min by distilled water (A) and a linear gradient of acetonitrile containing 0.1% TFA (B, 5–35%, in 30 min), and monitored at 220 nm. Each chromatographic run was repeated 15–20 times and the subfractions were pooled, freeze-dried, and subjected to ACE-inhibitory activity and ·OH scavenging activity assay. The fraction showing the highest activity was subjected to further isolation using RP-HPLC coupled with a Zorbax analysis C_18_ column (Φ 4.6 × 250 mm, Agilent Technologies, Palo Alto, CA, USA). The column was eluted with a linear gradient of solvent B (acetonitrile containing 0.1% TFA) in A (distilled water) going from 5% to 25% in 25 min at a flow rate of 1.2 mL/min. The fractions with high ACE-inhibitory activity and/or ·OH scavenging activity were subjected to liquid chromatography–mass spectrometry (LC-MS/MS) analysis.

### 2.7. Identification of Peptide Sequences

The peptide sequences were identified by LC-MS/MS coupled to an Eksigent Nano LC (Eksigent Technologies, Dublin, CA, USA) and Thermo LTQ linear ion trap mass spectrometer (Thermo Fisher, San Jose, CA, USA). Briefly, acetonitrile (2%, *v/v*) containing 0.1% formic acid was used as mobile phase A and acetonitrile (80%, *v/v*) with 0.1% formic acid as mobile phase B for nano-LC separation. Gradient elution was carried out according to the following process: phase B was linearly increased from 5% to 50% within 85 min; afterward, phase B was increased to 95% within the following 10 min, and then maintained at 95% for 30 min. Sequence identification of the eluted peptides was analyzed by MS/MS, and the parameters were as follows: spray voltage, 2.2 kV; normalized collision energy, 35%; capillary temperature, 200 °C; and scan range, *m/z* 300–2000. Peptide sequences were determined based on the acquired MS/MS data and analyzed by Xcalibur software (version 2.0.7, Thermo Fisher Scientific, Les Ulis, France) and Proteome Discoverer 2.1 (Thermo Fisher Scientific).

### 2.8. Screening for ACE-Inhibitory and Antioxidant Peptides by in Silico Approach and Peptide Synthesis

Databases including PepBank (http://pepbank.mgh.harvard.edu/) and BIOPEP (http://www.uwm.edu.pl/biochemia/index.php/en/biopep) were used to screen the peptides obtained from LC-MS/MS. Peptides with similarity to already identified ACE-inhibitory and/or antioxidant peptides were further subjected to chemical synthesis. Two peptide sequences predicted to have ACE-inhibitory activity through in silico approach were commercially synthesized at Yaoqiang Biotech Limited Co. (Shanghai, China) by standard solid-phase method. Purity of the peptides was more than 98%, and their antioxidant and ACE inhibition ability were measured.

### 2.9. Simulated Gastrointestinal Digestion of Synthesized Peptides

According to the method described by Gu et al. [24] with slight modification, the synthesized peptides were dissolved in potassium chloride solution (pH 2.0, adjusted by HCl) and digested by pepsin (E/S = 1:50) at 37 °C for 1.5 h. Afterward, the pH was increased to 7.4 and the sample was digested by pancreatin (E/S = 1:30) at 37 °C for 3 h, and then the digestion was terminated by boiling for 10 min. Then ACE-inhibitory activity of the treated synthetic peptides was determined in comparison to the activity of peptides before the digestion.

### 2.10. Molecular Modeling

The three-dimensional structure of ACE (1O8A.pdb) was downloaded from the Protein Data Bank (http://www.rcsb.org/pdb/home/home.do). Following the method presented by Ling et al. [25], all molecular modeling studies were performed on SYBYL-X 2.1.1. The structures of the purified peptides were constructed using the Build Protein function. A pocket was generated from 1O8A using the Dock Ligands function. All the water molecules and unwanted substructures were removed, and the essential hydrogen atoms were added. The purified peptides were docked into the pocket of ACE by molecular visualization. T-score (indicating the degree to which the inhibitors bind to ACE), hydrogen bond, and distance were calculated, and T-scores were accepted if their numerical value was higher than 6.0.

### 2.11. Antihypertensive Effect in Spontaneously Hypertensive Rats (SHRs)

Following the method indicated by Zou et al. [26] with some modifications, 20 male SHRs (10 weeks, 230 ± 20 g body weight (bw)) were purchased from Vital River Laboratory Animal Technology Co., Ltd (Beijing, China). After conditioning for one week, the rats were divided into five groups of four rats each. Physiological saline served as the negative control and captopril (14 mg/kg body weight once daily) was used as positive control. High dosage group (150 mg/kg body weight once daily), middle dosage group (100 mg/kg body weight once daily), and low dosage group (50 mg/kg body weight once daily) were orally given the peptide dissolved in 0.9% saline solution via gastric intubation. All of the treatments were conducted for five consecutive weeks. Each week, the systolic blood pressure (SBP) and diastolic blood pressure (DBP) of the rats were measured with a non-invasive blood-pressure apparatus (BP-300A Non-Invasive Blood Pressure Monitoring System, Chengdu, China). The body weight and heart rate of the rats were determined at the same time. At least five readings were recorded and averaged out. This experimental work was approved by the Institutional Animal Care and Use Committee, Shanxi Normal University (No. 20181211, 22 October 2018). All animals received humane care according to the Guide for the Care and Use of Laboratory Animals (National Institute of Health Publication No. 85–23, revised).

### 2.12. Antioxidant Activity of the Peptides

#### 2.12.1. ABTS Radical Scavenging Activity

Following the method proposed by Selamassakul et al. [27] with slight modifications, 20 mL of ABTS (7 mM) and 10 mL of K_2_S_2_O_8_ (7 mM) were mixed and incubated in the dark for 16 h to generate ABTS·^+^. The ABTS·^+^ solution was diluted with phosphate buffer (0.1 M, pH7.4) to reach an absorbance of 0.700 ± 0.010 at 734 nm. Afterward, 40 µL of sample solutions (100 mg/mL) and 4 mL of ABTS·^+^ solution were reacted at 30 °C for 6 min under darkness, and then the absorbance at 734 nm was recorded. The scavenging effect is expressed as the percentage of disappearance of the blue color of ABTS·^+^ at 734 nm. Glutathione (GSH) (1 mg/mL) was used as comparison.

#### 2.12.2. Metal Chelating Capacity

According to the method presented by Jeong et al. [28], a sample (100 µg/mL, 450 µL) was mixed with 45 µL of 2 mM FeCl_2_, 1815 µL of distilled water, and 90 µL of 5 mM ferrozine. The mixture was allowed to stand at room temperature for 30 min, and then the absorbance at 562 nm was read. GSH (100 µg/mL) was used as comparison and the chelating activity was calculated as follows:

(3) $$\mathrm{Chelating}\;\mathrm{activity}\;\left(\%\right){=\;[\;1-\;(A}_{S}{\;-\;A\;}_{B}{\;)/\;A}_{C}\;]\;\times 100,$$

where A_B_ is the absorbance of the blank, A_C_ is the absorbance of the control (without the addition of ferrozine), and A_S_ is the absorbance of the mixture-contained samples.

### 2.13. Statistical Analysis

All the experiments were repeated at least three times and mean values were used. Data were subjected to analysis of variance, and Duncan’s value with a confidence interval of 95% was calculated to compare the means.

## 3. Results and Discussion

### 3.1. ACE-Inhibitory and Antioxidant Activity of QBAH

The reason for using cellulase and hemicellulase in the current study was to increase the extraction ratio of quinoa bran protein, because they can hydrolyze the polysaccharides in cereals, break down the interaction, and crosslink between polysaccharides and proteins [20]. The subjection to hydrolysis by alcalase was followed by trypsin, and the hydrolysis degree of QBAH was 24.26% ± 3.61%, while the ACE inhibition activity and ·OH scavenging ability were 62.38% ± 5.64% and 51.77% ± 5.15%, respectively. It was reported that quinoa albumin had high levels of arginine, histidine, and lysine, and showed the maximum solubility at pH 9.0 [12]. Thus, the alcalase (with a suitable pH value of 8.5–10.0) was used to hydrolyze quinoa bran albumin in this study. Moreover, the trypsin was used to increase the stability of peptides against gastrointestinal digestion, as well as to improve the hydrolysis degree of QBAH. Nongonierma et al. [16] used papain to hydrolyze quinoa protein isolate but the DH was not mentioned, and the hydrolysates showed lower antioxidant activity (IC_50_ = 501.6 μM) than that of QBAH.

### 3.2. Isolation of the ACE-Inhibitory and Antioxidant Peptides 

**Figure 1:**
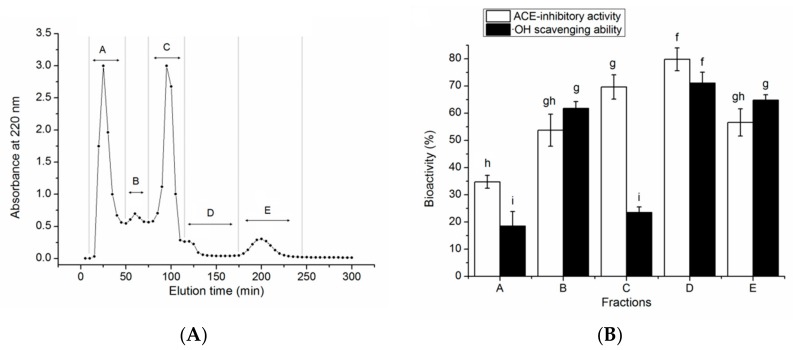
Purification profiles of quinoa albumin hydrolysates (QBAH) (**A**) by Sephadex G-25 gel chromatography and (**B**) ACE-inhibitory and antioxidant activity of each fraction. Different lowercase letters (f–i) on the bars mean significant difference (*p* < 0.05).

Figure 1 shows the filtration profile of QBAH using the Sephadex G-25 gel, as well as the ACE-inhibitory and antioxidant activity of each fraction.

**Figure 2:**
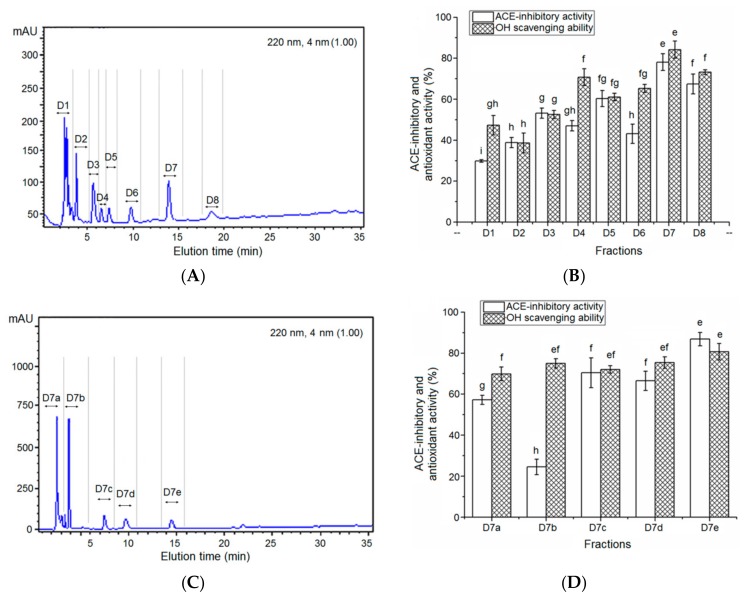
(**A**) RP-HPLC chromatography of fraction D on semi-preparing column and (**B**) ACE inhibition and antioxidant activity of each fraction. Separation was performed at a flow rate of 2.2 mL/min and a linear gradient width of 5–35% acetonitrile (containing 0.1% TFA) within 30 min. (**C**) Separation of fraction D7 on analytical RP-HPLC column and (**D**) ACE-inhibitory and antioxidant activities of fractions D7a, D7b, D7c, D7d and D7e. Elution was performed at a flow rate of 1.2 mL/min with a linear gradient width of 5–25% acetonitrile within 25 min. Different lowercase letters (e–h) on the bars mean significant difference (*p* < 0.05).

A total of five peaks named A to E were collected and significant improvement in antioxidant and ACE-inhibitory activity of QBAH was obtained after the gel chromatography. Of these, the fraction D showed the highest ACE-inhibitory activity (79.78% ± 4.17%) and ·OH scavenging ability (71.04% ± 4.05%). Thus, it was applied to RP-HPLC separation with a semi-preparative column on the basis of non-polarity and the elution profile is shown in Figure 2.

As shown in Figure 2A, about eight major peaks named D1 to D8 were present, collected, and used to measure ACE-inhibitory and antioxidant activity at a concentration of 1 mg/ mL. The fraction D7 demonstrated the highest ACE-inhibitory activity (78.08% ± 4.07%) and ·OH scavenging capacity (84.17% ± 4.21%) among these fractions (Figure 2B). Thus, it was further purified by RP-HPLC on the analytical C_18_ column (Φ 4.6 × 250 mm) and separated into five major fractions (D7a, D7b, D7c, D7d, and D7e, Figure 2C). Of these, the fraction D7e showed the highest ACE-inhibitory activity (86.83% ± 3.24%, at 1 mg/mL) and ·OH scavenging capacity (84.69% ± 2.79%, at 1 mg/mL, Figure 2D). Therefore, it was chosen for ESI-MS/MS analysis.

### 3.3. Characterization of Pooled Peptide Fraction by Mass Spectrometry and Screening of ACE-Inhibitory Peptides

**Figure 3:**
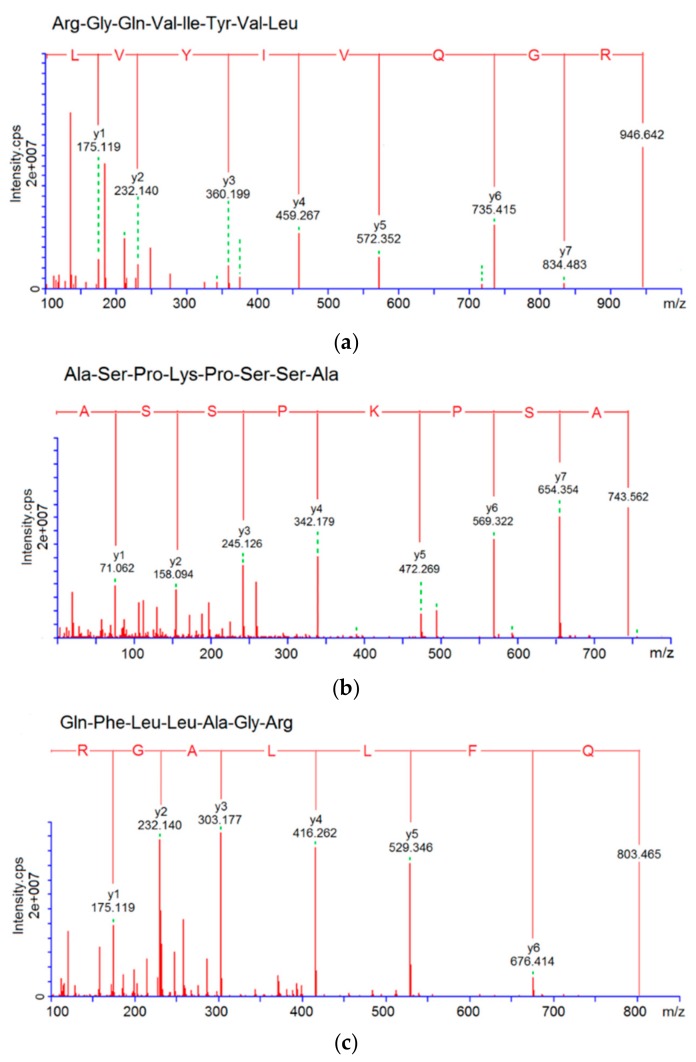
ESI-MS/MS spectrum analysis of peptides (**a**) RGQVIYVL, (**b**) ASPKPSSA, and (**c**) QFLLAGR.

**Figure 4:**
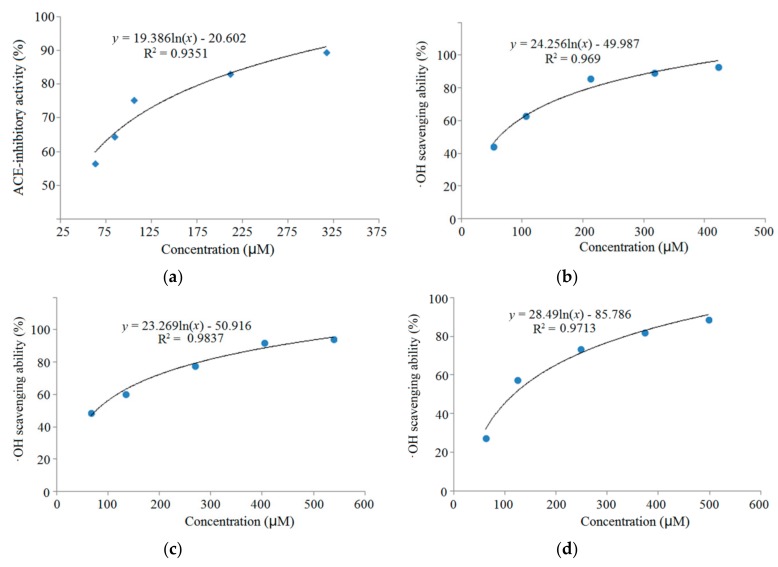
The ACE-inhibitory and antioxidant activity and the regression analysis of (**a**,**b**) RGQVIYVL, (**c**) ASPKPSSA, and (**d**) QFLLAGR.

Results of the LC-MS/MS analysis showed that the fraction D7e was composed of twelve peptides (Table 1). With the help of databases AHTPDB and BIOPEP, and based on the similarity to the already reported ACE-inhibitory and antioxidant peptides, three peptides, namely RGQVIYVL (946.6 Da), ASPKPSSA (743.8 Da), and QFLLAGR (803.5 Da), were chosen for chemical synthesis. Their mass spectra by nano-LC-ESI-MS/MS are shown in Figure 3a–c. Their molecular weight, amino acid sequence, and the relationship between the ACE-inhibitory activity or ·OH scavenging activity (*y*) of the peptides and the concentrations (*x*) are shown in Table 1 and Figure 4. As shown in Table 1, only RGQVIYVL demonstrated high ACE-inhibitory activity (IC_50_ = 38.16 μM) and ·OH scavenging activity (IC_50_ = 61.69 μM); on the other hand, ASPKPSSA, and QFLLAGR only showed ·OH scavenging ability (IC_50_ = 76.47 and 117.46 μM, respectively). Results in Figure 4A demonstrated that the regression equation of RGQVIYVL was *y* = 19.386 ln(*x*) − 20.602 (R^2^ = 0.9351), according to which the IC_50_ value of ACE-inhibitory activity was calculated to be 38.16 μM. This value is much higher than that of captopril (0.023 μM) [6]. Moreover, the regression equations of RGQVIYVL, ASPKPSSA, and QFLLAGR were *y* = 24.256 ln(*x*) − 49.987 (R^2^ = 0.969), *y* = 23.269 ln(*x*) − 50.916 (R^2^ = 0.9837), and *y* = 28.49 ln(*x*) − 85.786 (R^2^ = 0.9713) (Figure 4B–D), respectively. Based on the above equations, the IC_50_ values of ·OH scavenging activity of RGQVIYVL, ASPKPSSA, and QFLLAGR were calculated as 61.69, 76.47, and 117.46 μM, respectively. Moreover, they were all novel peptides identified in quinoa not previously reported.

It was obvious that the three peptides were oligopeptides with 7 or 8 amino acid residues and rich in hydrophobic amino acids. Moreover, RGQVIYVL and QFLLAGR were rich in branched amino acids (Val, Leu, and Ile) and RGQVIYVL contained aromatic amino acids (Tyr) in C-terminal tripeptide. The above results further confirmed the report that the majority of ACE-inhibitory and antioxidant peptides are short sequences with 2–12 residuals, of which hydrophobic amino acids (Phe, Val, Leu, Ile, Pro, Ala, Trp, and Met) are the major ones [10,29].

Although the structural bioinformatics showed that ACE-inhibitory activity had a negative correlation with molecular mass of peptides, RGQVIYVL (946.6 Da) showed a higher activity than that of peptides identified from tilapia skin gelatin (VGLPNSR, IC_50_ = 80.9 μM), horse gram protein (QLLLQQ, IC_50_ = 75.0 μM), and rice bran protein (YSK, IC_50_: 76 μM) [25,30,31]. The reason may be the presence of Tyr and branch amino acids (Val and Leu) at the C-terminal of RGQVIYVL simultaneously. In recent years, it has been demonstrated that ACE appears to prefer substrates or competitive inhibitors that contain hydrophobic amino acid residues, particularly Val, Leu, and/or aromatic residues (Trp, Tyr, or Phe) in the C-terminal tripeptide [25,29]. Moreover, accumulating studies suggest that the positive charge of Arg (guanidine group) at N-terminus contributes substantially to the inhibitory activity against ACE [32,33].

Regarding the antioxidant activity of peptides, it was more related to size, amino acid constituents and sequence, structure, and hydrophobicity [27]. A previous study on the structure–activity relationship of peptides demonstrated that some amino acids with special structures such as aromatic amino acids and Arg, His, Pro, and Lys were important to the antioxidant property [3]. Tyr and Phe had the special capability of phenolic groups to serve as hydrogen donors; Trp had the capacity of the indolic group to serve as hydrogen donor [4]. Moreover, the guanidine group of Arg, imidazole group of His, and pyrrolidine group of Pro all had the proton-donation ability, attributed to the antioxidant capacity of peptides [10,34]. Therefore, the antioxidant activity found in RGQVIYVL, ASPKPSSA, and QFLLAGR was probably attributed to the presence of Tyr, Phe, Pro, Arg, Val, Ile, and Leu. RGQVIYVL demonstrated a higher antioxidant activity than that of ASPKPSSA, and QFLLAGR, probably due to the high content of hydrophobic amino acids (62.50%, Table 1) and branch amino acids (Val, Ile, and Leu), as well as the presence of Gln residue [34,35].

### 3.4. Stability Against in Vitro Digestion of Synthetic Peptides

**Figure 5:**
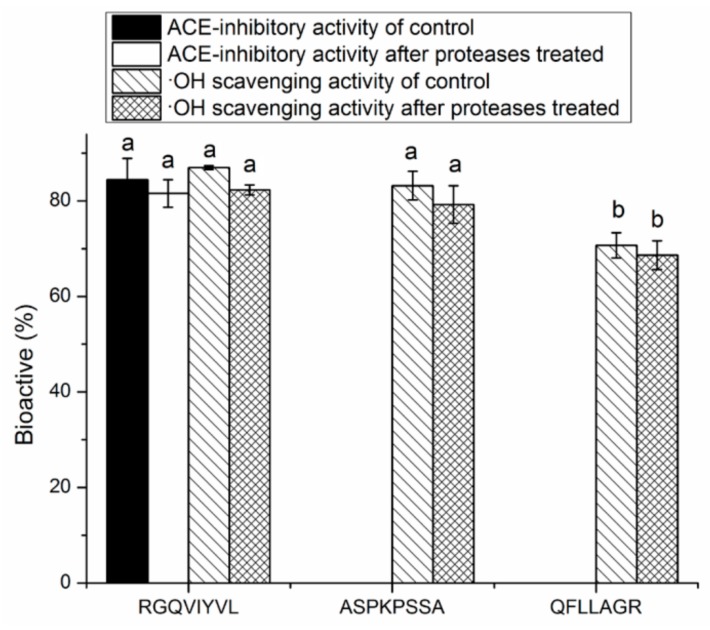
Stability of synthetic peptides RGQVIYVL, ASPKPSSA, and QFLLAGR against the simulated gastrointestinal digestion (hydrolyzed with pepsin followed by pancreatin). Different lowercase letters (a,b) on the bar mean significant difference (*p* < 0.05).

It was demonstrated that ACE-inhibitory peptides must be absorbed from the intestine and keep the peptide chains relatively intact before entering into the blood circulation to exert antihypertensive or other effects [11]. As shown in Figure 5, RGQVIYVL, ASPKPSSA, and QFLLAGR all showed similar levels of ACE-inhibitory and/or antioxidant activity before and after the digestion, suggesting that they could effectively retain the activity in the gastrointestinal digestion system. It was reported that pancreatin preferentially hydrolyzes peptide bonds with aromatic side chains (Tyr, Trp, and Phe) near N-terminal or C-terminal, and pepsin has preferential cleavage for bonds after uncharged non-branched residues such as Phe, Leu, and Tyr [34]. RGQVIYVL and QFLLAGR with Tyr or Phe residues could keep the activity after the digestion, perhaps because the peptides break into smaller peptides with similar activity [36].

### 3.5. Inhibition Kinetics of Synthetic Peptides

**Figure 6:**
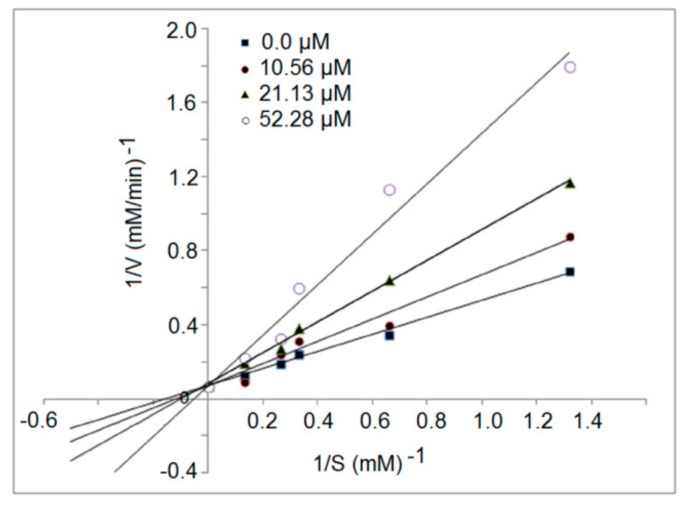
Lineweaver–Burk plots of the ACE inhibition for peptide RGQVIYVL.

The inhibition mode of the synthetic peptides was evaluated by a Lineweaver–Burk plot. The kinetic constants of ACE in the presence of peptide RGQVIYVL revealed that the Michaelis–Menten constant maximum velocity (*V_max_*) of the reaction remain unchanged, whereas the (*K_m_*) decreased as the inhibitor concentration increased, which is characteristic of competitive inhibition modalities (Figure 6). This result suggests that these inhibitor peptides could bind to the active site of ACE [25]. Over the last few years, many ACE inhibition peptides have been identified from food-derived proteins, most of which belong to the competitive mode [6]. Moreover, more peptides with uncompetitive or mixed inhibition manners have also been found in recent years [31,37].

### 3.6. Molecular Docking Simulation between the Peptides and ACE

**Figure 7:**
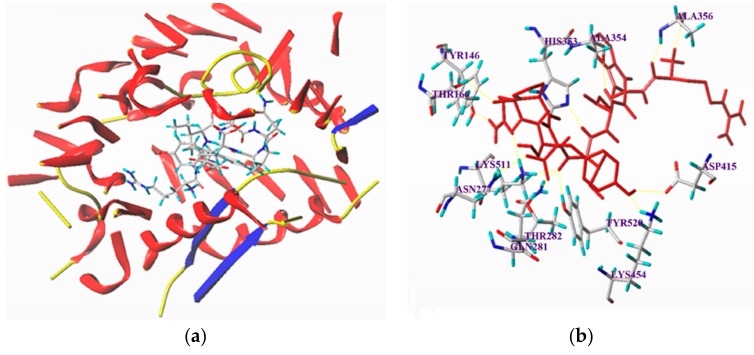
General overview (**a**) and local overview (**b**) of the best-ranked docking pose of RGQVIYVL binding with ACE (PDB: 1O8A).

Molecular docking studies are effective in finding the relationships between the structure and function of peptides. In this study, the molecular modeling software SYBYL-X 2.1.1 was used to obtain insights into the binding mode of RGQVIYVL into ACE. As shown in Figure 7 and Table 2, the docking simulation of the ACE–ligand complexes was well performed between ACE and RGQVIYVL. T-scores represent the binding affinities of ligand and protein enzyme, and the high T-score values indicated the strong binding power [25]. After docking, the T-score of RGQVIYVL was 10.66, which was much higher than 6.0 (the acceptable critical value of the T-score), indicating the strong binding power. It was demonstrated that hydrogen bond interaction force plays an important role in stabilizing the docking complex and enzyme catalytic reactions [33]. As shown in Table 2, RGQVIYVL showed 13 hydrogen bonds with ACE residues, and the distances of the hydrogen bonds to the amino acid residues were very short, also indicating that the peptide bonding with the ACE was strong [25]. These data suggest that RGQVIYVL could interact effectively with ACE, mainly attributed to its high ACE-inhibitory activity (Table 1).

Previous studies have shown that ACE has three main active site pockets (S1, S2, and S1′). S1 pocket includes Ala354, Glu384, and Tyr523 residues and S2 pocket includes Gln281, His353, Lys511, His513, and Tyr520 residues, whereas S1′ contains Glu162 residue [38]. Moreover, other key amino acid residues, namely Glu411, Glu162, His383, and His387, were also present in the ACE active sites [39]. Results in Figure 6B demonstrated that RGQVIYVL could form hydrogen bonds with Asp415, Lys454, Tyr520, Thr282, Gln289, Asn277, Lys511, Thr166, Tyr146, His353, Ala354, and Ala356. Of these, Tyr520, Lys511, and His353 belong to the key residues in the ACE active site S2, and Ala354 was in the ACE active site S1. These results revealed that RGQVIYVL could inhibit ACE activity through interacting with the key active site of ACE, in accordance with its determined ACE inhibition kinetic pattern (Figure 5). Some peptides such as YSK (bonding with Ala354, Gln281, and His353) and LPLPLL (bonding with Glu411,33) were also competitive [31].

### 3.7. Antihypertensive Effect of Peptides on SHRs

**Figure 8:**
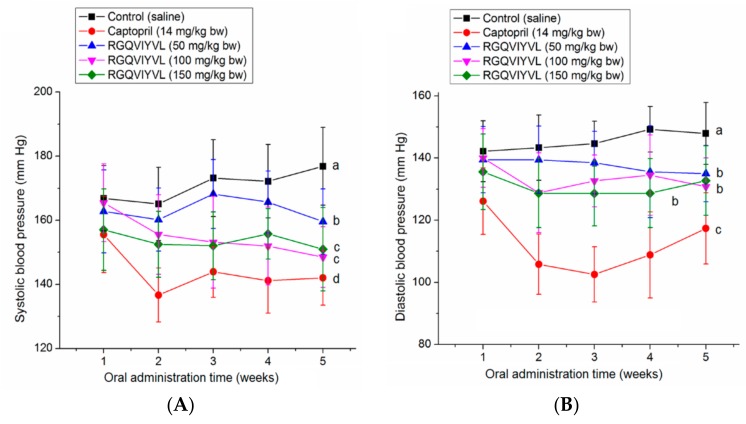
Effects of oral administration of individual peptide RGQVIYVL on (**A**) diastolic blood pressure (DBP) and (**B**) systolic blood pressure (SBP) of spontaneous hypertensive rats (SHRs). SHRs in low-, middle- and high-dose groups were orally administered peptide at 50, 100, and 150 mg/kg body weight (bw) every day, respectively. SHRs of the positive control group were given captopril at 14 mg/kg body weight once daily, whereas the SHRs in the control group were only given physiological saline (0.5 mL). Different lowercase letters near the lines (a–c) mean significant difference (*p* < 0.05).

As shown in Figure 8A,B, oral administration of RGQVIYVL at different concentrations (100–150 mg/kg body weight) all caused a significant decrease in both DBP and SBP of SHRs from the second week (*p* < 0.05). However, there was no significant dose-dependent relationship between the high- and medium-dose groups, which may be due to the compensatory activation of other systems (e.g., nervous system, endothelin system, and nitric oxide system) involved in blood pressure control [27]. The results suggested that RGQVIYVL had antihypertensive effect on SHRs, although it was weaker than that of captopril. In addition, results in Supplementary Materials Tables S1 and S2 demonstrated that neither heart rate nor body weight of SHRs in all sample groups was significantly different from that in the negative control group (*p* > 0.05), indicating that there was no obvious side effect on SHRs during the administration of RGQVIYVL.

### 3.8. Antioxidant Activity of the Synthetic Peptides

#### 3.8.1. Radical Scavenging Activity

**Figure 9:**
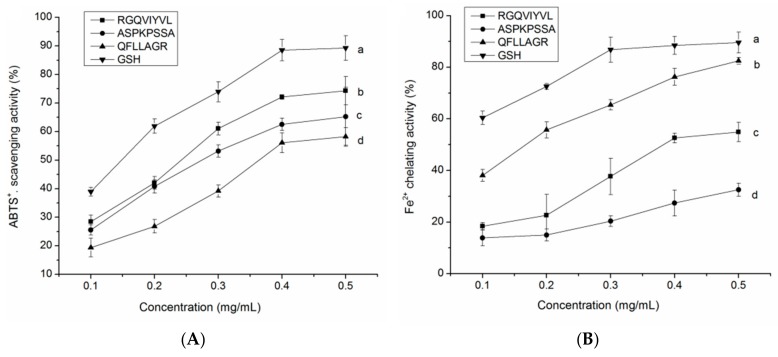
The ABTS·^+^ scavenging activity (**A**) and Fe^2+^ chelating ability (**B**) of peptides RGQVIYVL, ASPKPSSA, and QFLLAGR at different concentrations. Glutathione (GSH) was used as the positive control. Lowercase letters near the line (a–d) mean significant difference (*p* < 0.05).

ABTS·^+^ scavenging activity represents the total antioxidant activity of potential antioxidants [38]. As depicted in Figure 9A, the three peptides identified in QBAH all exhibited remarkable ABTS·^+^ scavenging activity with a concentration-dependent increase, indicating that they had considerable total antioxidant activity. The highest scavenging activity (74.29% ± 4.97%, at 0.5 mg/mL) was found in peptide RGQVIYVL, probably attributed to its higher branch amino acids (Val, Ile, and Leu) and proton-donor amino acids (Thr, Gly, Gln, and Arg) [34]. Moreover, the presence of hydrophobic amino acids (Ala, Pro, Phe, and Ile) contributed to the high ABTS·^+^ scavenging activity of ASPKPSSA, and QFLLAGR. These amino groups can easily donate protons to electron-deficient radicals while maintaining their stability via resonance structures [27,40]. Furthermore, the ABTS·^+^ scavenging ability of peptides identified in the current study was higher than that of VTSLDLPVLRW (IC_50_ = 661 μM) and HPLDSLCL (IC_50_ = 74.36 μM) [41,42].

#### 3.8.2. Fe^2+^ Chelating Ability

The presence of some metal ions like Fe^2+^ and Cu^2+^ can extremely accelerate the oxidative chain reaction of oil and induce the production of more various radicals [38]. As shown in Figure 9B, RGQVIYVL, ASPKPSSA, and QFLLAGR all showed a relatively high Fe^2+^ chelating ability (32.54–82.48% at 0.5 mg/mL), which was lower than that of GSH. QFLLAGR showed the highest chelating ability among the three peptides, probably attributed to its lower molecular weight and the residues Phe, Gln, and Arg. It was pointed out that amino acid residues such as Pro, Phe, and Arg in the side chain could act as metal ion chelator [27]. Moreover, the chelating ability of QFLLAGR was also much higher (*p* < 0.05) than that of peptides identified from sweet potato protein (YYIVS, 59.74% at 3 mg/mL) and tilapia gelatin (Leu-Ala-Arg-Leu, 46.53% at 1 mg/mL) [38,43]. This result indicated that the peptides identified from QBAH could terminate the free radical chain reaction by clearing its catalyst (metal ions like Fe^2+^).

## 4. Conclusions

One ACE-inhibitory and antioxidant peptide, RGQVIYVL (946.6 Da), and two antioxidant peptides, ASPKPSSA (743.8 Da), and QFLLAGR (803.5 Da), were identified in QBAH. RGQVIYVL showed a high ACE-inhibitory activity (IC_50_: 38.16 μM) with competitive mode of inhibition, and showed significant antihypertensive effect in spontaneously hypertensive rats at the concentration of 100 to 150 mg/kg body weight. Molecular docking simulation showed that it could interact with the active ACE site via 13 hydrogen bonds. Moreover, RGQVIYVL, ASPKPSSA, and QFLLAGR all demonstrated high ·OH scavenging activity (IC_50_ = 61.69–117.46 μM), ABTS^+^ scavenging activity (58.29–74.28%) and Fe^2+^ chelating ability (32.54–82.48%). These results highlighted the future application of quinoa albumin in the nutraceuticals field. However, their bioavailability in vivo should be studied in future work.

## Figures and Tables

**Table 1:** Peptide sequences identified in fraction D7e by LC-MS/MS and their ACE-inhibitory and antioxidant activity ^a^.

Peptides	Molecular Mass (Da)	Matched Sequence in *Chenopodium quinoa* Willd. ^a^	Calculated pI	Hydrophobic Residue Content	IC_50_ (μM) of ACE-Inhibitory Activity	IC_50_ (μM) of ·OH Scavenging Ability
NGGGGGGGSGGAH	941.4	G.NGGGGGGGSGGAH.A	5.67	15.38%		
GEHMAGGS	744.8	I.GEHMAGGS.S	6.76	22.22%		
RGQVIYVL	946.6	R.RGQVIYVL.G	4.98	62.50%	38.16	61.69
LGGGAGGGGGIGGG	943.5	R.GREEEEGR.G	9.06	21.43%		
SKIGEHMA	872.0	G.SKIGEHMA.G	6.76	37.50%		
ASPKPSSA	743.8	T.ASPKPSSA.S	8.02	50.00%		76.47
SGGSGAG	491.4	P.SGGSGAG.P	5.33	14.29%		
AGGGGGYGAGG	651.6	A.GGGGGYGAG.G	5.90	18.18%		
DQGAGYGGG	780.7	G.DQGAGYGGG.G	5.90	11.11%		
EAGGGEGGGGGEGG	1046.9	Q.EAGGGEGGGGGEGG.G	5.97	7.14%		
QFLLAGR	803.5	G.QFLLAGR.G	8.16	30.00%		117.46
QGAGYGGGGGSGG	980.9	D.QGAGYGGGGGSGG.G	5.90	7.69%		

^a^ From the National Center for Biotechnology Information (NCBI).

**Table 2:** Docking scores and hydrogen bonds observed between peptides and ACE from molecular docking simulation.

Ligand	T-Score	Hydrogen Bond Number	Distance (Å)
RGQVIYVL	10.66	13	ASP415: 2.49; Ala356: 1.81,1.99; Ala354: 2.05; His353: 2.89; Tyr 146: 1.64; Lys511: 2.22, 1.89; Tyr520: 2.10; Gln281: 2.04; Thr282: 1.73; Asn277: 2.73; Lys454: 1.90

## Supplementary Materials

The following are available online at https://www.mdpi.com/1420-3049/24/24/4562/s1, Table S1: Body weight of the spontaneous hypertensive rats administrated with peptide RGQVIYVL, Table S2: Heart rate of the spontaneous hypertensive rats administrated with peptide RGQVIYVL.
