# Supplementary material for: Isolation of Novel ACE-Inhibitory and Antioxidant Peptides from Quinoa Bran Albumin Assisted with an In Silico Approach: Characterization, In Vivo Antihypertension, and Molecular Docking
Source: Molecules. 2019 Dec 12;24(24):4562. doi: 10.3390/molecules24244562 (PMC6943578; doi:10.3390/molecules24244562)
Supplement: Supplementary file 1 [file molecules-24-04562-s001.pdf]

molecules

MDPI

Supplementary

Table S1. Body weight (g) of the spontaneous hypertensive rats administrated with peptide RGQVIYVL [a]

Time / Week	Negative control				Captopril control				Low dose group				Middle dose group				High dose group			
0	22	22	22	21	23	22	21	21	21	20	19	23	23	22	21	20	23	21	22	21
	7.9	5.2	1.7	3	1.8	2.7	9.3	1.8	2.1	3	5.6	8	8.5	6.8	6.1	7.1	3.4	9.2	8.9	7.9
1st	25	23	24	23	25	24	23	23	23	21	21	24	24	24	22	23	24	22	23	23
	3.1	7.7	8.3	6.4	6.9	5.8	1.6	0.8	1.7	6.9	8.6	9.6	9.2	4.3	0.9	4	5.9	9.5	6.8	2.4
2nd	26	24	26	25	26	26	23	24	23	22	22	26	26	25	23	24	25	24	24	24
	6	8.3	2.1	1.4	7.9	0.7	7.7	0.9	8.2	8	9.4	0.7	2.1	8	1.8	6.3	7.1	4	2.3	2.8
3rd	28	26	27	26	27	26	24	24	25	23	23	26	27	27	24	26	25	24	25	24
	2.4	8.1	3.4	6.7	6.4	2.5	2.4	7.5	0.2	7	7.4	5.3	5.8	4.7	0.7	2.1	7	9.2	1.5	8.1
4th	28	27	28	27	28	28	26	25	26	24	24	26	29	28	24	27	27	26	26	25
	8.3	9.5	2.1	2.7	3.1	1	5.2	7.9	0.6	6.9	6.7	9.4	0	1.2	8.8	6.4	6.8	4.7	5	7.4
5th	30	28	30	28	28	29	27	26	25	25	24	27	29	28	25	28	28	27	27	26
	2.7	9.4	0.7	1.4	9.2	7.3	1.8	4.1	7.5	1.8	7.2	3.5	4.9	9.4	7	0.2	7.9	6.9	5.6	2.8

[a] Rats in Low-, middle- and high- dose groups were orally given the peptide at 50, 100 and 150 mg/kg/bodyweight once daily, respectively.

Table S2 Heart rate of the spontaneous hypertensive rats administrated with peptide RGQVIYVL [a]

Groups	Heart rate																	
	The first week																	
Negative	5.	5.	6.	6.	6.	6.4	6.	6.	6.	7.	8.2	6.	5.	7.	6.			
control	8	87	78	69	92	1	43	45	67	92	2	67	83	59	77			
Captopril	5.	7.		5.	6.	6.9	7.	6.	6.	7.	6.5	6.						
control	51	12	6	81	8	5	52	65	14	41	7	49						
Low dose	7.	6.	6.	6.	6.	6.5	6.	7.	6.	9.	7.9							
group	14	71	35	86	99	2	88	32	55	55	8							
Middle	7.		7.	6.	6.	9.6	7.	8.	6.	7.	7.2	7.	8.		8.			
dose group	4	7	05	19	65	9	67	61	69	06	7	78	13	8	59			
High dose	8.	7.	7.	7.	6.	8.2	8.	6.	6.	6.	7.2	7.						
group	63	87	56	23	34	2	62	5	38	25	8	41						
	The second week																	
Negative	6.	6.	6.	6.	6.	10.	7.	5.	6.	7.	7.3	7.	8.	7.	6.	6.	8.	8.
control	45	93	74	78	45	52	3	82	68	48	7	82	19	29	57	81	63	71
Captopril	7.	8.	8.	7.	6.		9.	6.	9.	8.	11.	9.						
control	24	28	42	63	9	7	12	19	25	43	35	81						
Low dose	6.	8.	8.	8.		7.3	6.	6.	5.	7.		7.						
group	28	82	87	97	8	4	12	15	83	29	7	68						
Middle	7.	7.	7.	7.	7.	7.1	6.	7.	7.	6.	5.8							
dose group	25	76	95	57	08	4	77	07	59	69	7							
High dose	6.	5.	5.	5.	5.	6.6	6.	6.	6.	6.		5.	6.	6.				
group	39	94	93	69	63	1	97	74	44	51	5.8	87	92	78				
	The third week																	

Negative	8.	7.	8.	6.	6.	7.1	8.	6.	7.	8.	8.6	8.	6.				
control	1	08	19	19	34	4	22	28	61	57	4	6	97				
Captopril	6.	5.	5.	7.	7.	7.1	6.	6.	6.	7.	7.2	7.	6.				
control	92	91	61	28	91	8	11	04	27	53	9	63	48				
Low dose	8.	7.	6.	5.	6.		7.	7.	7.	7.	7.2	8.	8.	8.			
group	04	14	57	83	25	6.7	3	19	19	41	6	44	7	06			
Middle	6.	6.	6.	7.	7.	8.2	7.	6.	7.	6.	7.5	6.					
dose group	48	1	24	09	47	1	38	87	01	5	2	63					
High dose	8.	6.	7.	8.	8.		8.	7.	8.	6.	6.3	7.	6.				
group	22	28	61	57	64	8.6	1	08	19	19	4	14	97				
								The fourth week									
Negative	6.	7.	8.	6.			8.	6.	7.	6.		5.	5.	5.			
control	58	02	09	83	8	6.9	33	23	91	14	7.1	67	8	91			
Captopril	5.	6.	6.	6.		6.1	5.	5.	6.	7.	8.3	7.	7.				
control	88	67	52	64	6	9	39	61	57	41	3	63	86				
Low dose	7.	6.	7.		5.	5.6	5.	6.	7.	5.	6.8	7.	7.				
group	06	86	3	8	83	3	73	6	76	68	2	78	82				
Middle	6.	6.	6.	6.	6.	6.4	6.	7.	7.	6.	6.3	6.	7.				
dose group	49	33	53	35	26	8	51	63	21	44	4	9	79				
High dose	6.	7.	5.	6.	6.	5.8	6.	7.	6.	6.	6.4	5.					
group	64	14	99	93	57	3	25	41	57	49	4	68					
								The fifth week									
Negative	6.	6.	6.	6.	6.	6.3	6.	6.	6.	6.	6.8	6.	6.	6.	6.		
control	08	19	43	14	46	8	45	64	95	85	4	5	58	54	58		
Captopril	5.	5.	6.	5.	5.	5.6	6.	6.	6.	6.	7.2	5.	5.	5.	6.	6.	6.
control	53	75	39	94	93	9	51	92	74	78	7	51	5	92	06	22	98
Low dose	6.	7.	6.	7.	6.	6.0	6.	6.	6.	6.	7.2	6.	7.				
group	81	18	71	14	05	7	67	79	91	86	5	88	11				
Middle	6.	6.	6.	6.	6.	6.8	6.	6.	5.	6.	6.6	6.					
dose group	39	37	15	9	44	5	81	94	91	63	2	88					
High dose	7.	6.	6.	6.	6.	5.9	7.	6.	6.	6.	7.4						
group	63	19	36	74	42	8	72	71	6	92	1						

[a] Rats in Low-, middle- and high- dose groups were orally given the peptide at 50, 100 and 150 mg/kg/bodyweight once daily, respectively.
